# Supplementary material for: Road properties of cement-phosphogypsum-red clay under dry and wet cycles
Source: PLoS One. 2024 Dec 19;19(12):e0314276. doi: 10.1371/journal.pone.0314276 (PMC11658611; doi:10.1371/journal.pone.0314276)
Supplement: S1 Dataset — (DOCX) [file pone.0314276.s001.docx]

**Minimum data set**

**Table.1 Basic physical indexes of red clay**

| $\boldsymbol{\rho}$/g‧cm^-3^ | $\boldsymbol{\omega}$/% | $\boldsymbol{\omega}_{\boldsymbol{op}}$/% | $\boldsymbol{\rho}_{\boldsymbol{dmax}}$/g‧cm^-3^ | $\boldsymbol{W}_{\boldsymbol{L}}$/% | $\boldsymbol{W}_{\boldsymbol{P}}$/% | Cu | Cc |
| --- | --- | --- | --- | --- | --- | --- | --- |
| 1.76 | 60.03 | 30.24 | 1.46 | 82.13 | 43.02 | 10.63 | 1.085 |

**Table.2 Chemical Composition of Red Clay**

| Si/% | Fe/% | Al/% | K/% | Mg/% | O/% |
| --- | --- | --- | --- | --- | --- |
| 25.84 | 8.02 | 14.75 | 2.68 | 0.88 | 43.56 |
| SiO_2_/% | Al_2_O_3_/% | Fe_2_O_3_/% | K_2_O/% | MgO/% | TiO_2_/% |
| 55.60 | 27.32 | 11.26 | 3.12 | 1.39 | 1.20 |

**Table.3 Basic Parameters of Phosphogypsum**

| Specific surface area  / m^2^‧kg^-1^ | Loss on ignition /% | Moisture content /% | Alkali content /% | Density  /g‧cm^-3^ | fineness  /% |
| --- | --- | --- | --- | --- | --- |
| 102 | 18.43 | 5.3 | 1.31 | 2.38 | 44.3 |

**Table.4 Chemical Composition of Phosphogypsum**

| Ingredient | SO_3_ | CaO | SiO_2_ | P_2_O_5_ | Na_2_O | Al_2_O_3_ | Other |
| --- | --- | --- | --- | --- | --- | --- | --- |
| Mass fraction/% | 49.070 | 40.070 | 5.780 | 1.350 | 0.587 | 0.435 | 2.708 |

**Table.5 Test Results of Heavy Metals and Radioactivity in Phosphogypsum**

| Test items | | Standard limits | Result | Conclusion |
| --- | --- | --- | --- | --- |
| Heavy metal | Cu/mg‧L^-1^ | ≤100 | 0.157 | Qualified |
|  | Zn/mg‧L^-1^ | ≤100 | 0.051 | Qualified |
|  | Cd/mg‧L^-1^ | ≤1 | 0 | Qualified |
|  | Pb/mg‧L^-1^ | ≤5 | 0 | Qualified |
|  | Cr/mg‧L^-1^ | ≤15 | 0 | Qualified |
|  | As/mg‧L^-1^ | ≤5 | 0.0356 | Qualified |
|  | Hg/mg‧L^-1^ | ≤0.1 | 0.0005 | Qualified |
| Radioactivity | Ra-226/Bq‧kg^-1^ | — | 53.94 | — |
|  | TH-232/Bq‧kg^-1^ | — | 42.13 | — |
|  | K-40/Bq‧kg^-1^ | — | 52.95 | — |
|  | I_Ra_ | ≤1.0 | 0.3 | Qualified |
|  | I_γ_ | ≤1.0 | 0.3 | Qualified |

**Table.6 Basic parameters of cement**

| Item | Index | Item | Index | Item | Index |
| --- | --- | --- | --- | --- | --- |
| 3d $f_{cf}$/MPa | 5.0 | Loss on ignition /% | 1.58 | Initial setting time /min | 302 |
| 28d $f_{cf}$/MPa | 6.7 | Alkali /% | 2.42 | Final setting time /min | 322 |
| 3d $f_{cu}$/MPa | 24.9 | Chloride ion /% | 0.018 | Stability | Qualified |
| 28d $f_{cu}$/MPa | 43.7 | Sulfur trioxide /% | 2.87 |  |  |

**Table.7 SCA-2 curing agent basic parameters**

| Morphology of an object | $\boldsymbol{\rho}$ /g.cm-3 | Stickiness /MPa·S | PH value |
| --- | --- | --- | --- |
| Fluids | 1.06 | 180 | 6.0-8.0 |
| Self-accelerating decomposition temperature /℃ | Solubility | Characteristics of the object | Combustion point |
| 60 | Water-soluble | Non-toxic, non-corrosive, non-polluting | Non-combustible |

**Table.8 Mixing ratios**

| C/% | P/% | T/% | K/% | Optimum moisture content/% | Dosage of water stabiliser/% |
| --- | --- | --- | --- | --- | --- |
| 5 | 47.5 | 47.5 | 96 | 22.9 | 0 |
|  |  |  |  |  | 5 |

**Table.9 C: P: T=5: 47.5: 47.5, Numerical value of unconfined compressive strength without water stabilizers/ MPa**

| Number of wet and dry cycles/N | 7d curing period | 14d curing period | 28d curing period |
| --- | --- | --- | --- |
| 0 dry wet cycles | 1.51 | 2.33 | 2.67 |
| 1 dry wet cycle | 1.22 | 1.61 | 1.88 |
| 2 dry wet cycles | 0.69 | 1.18 | 1.48 |
| 3 dry wet cycles | 0.66 | 0.73 | 1.02 |
| 4 dry wet cycles | 0.58 | 0.62 | 0.87 |
| 5 dry wet cycles | 0.31 | 0.41 | 0.53 |

**Table.10 C: P: T=5: 47.5: 47.5, Unconfined compressive strength values of water-doped stabilizers/ MPa**

| Number of wet and dry cycles/N | 7d curing period | 14d curing period | 28d curing period |
| --- | --- | --- | --- |
| 0 dry wet cycles | 0.92 | 1.21 | 1.35 |
| 1 dry wet cycle | 0.71 | 0.92 | 1.07 |
| 2 dry wet cycles | 0.61 | 0.73 | 0.81 |
| 3 dry wet cycles | 0.45 | 0.61 | 0.69 |
| 4 dry wet cycles | 0.42 | 0.53 | 0.62 |
| 5 dry wet cycles | 0.38 | 0.47 | 0.54 |

**Table.11 Table of water stability coefficients of cement phosphogypsum stabilised red clay mixes**

| mixing ratio | K/% | Number of dry and wet cycles | coefficient of water stability/% |
| --- | --- | --- | --- |
| C：P：T=5：47.5：47.5，Unmixed water stabiliser | 96 | 0 | 0 |
|  |  | 1 | 0 |
|  |  | 2 | 0 |
|  |  | 3 | 0 |
|  |  | 4 | 0 |
|  |  | 5 | 0 |
| C：P：T=5：47.5：47.5，Addition of 5% SCA-2 water stabiliser | 96 | 0 | 84.52 |
|  |  | 1 | 55.55 |
|  |  | 2 | 38.88 |
|  |  | 3 | 0 |
|  |  | 4 | 0 |
|  |  | 5 | 0 |

**Table.12 Quantitative analysis table of mix composition**

| Mineral phase content/ wt% | C: P: T=5: 47.5: 47.5, Unmixed water stabiliser | C: P: T=5: 47.5: 47.5, Addition of water stabiliser |
| --- | --- | --- |
| SiO_2_ | 4.9 | 11.6 |
| CaCO_3_ | - | 1.1 |
| CaSO_4_ • 2H_2_O | 86.5 | 59.3 |
| FeO • OH | 0.8 | 4.0 |
| (K, Na)Al_2_(Si, Al) _4_O_10_(OH)_2_ | 3.5 | 10.4 |
| Al_2_(OH) _4_Si_2_O_5_ | 2.5 | 12.0 |
| Ca_6_Al_2_ (SO_4_)3 (OH)_12_ • 26H_2_O | 1.8 | 1.6 |

**Tab.13 Mix CBR value/%**

| Compaction degree | 7d curing period | 14d curing period | 28d curing period | Note |
| --- | --- | --- | --- | --- |
| 96% compaction degree | 25.1 | 45.9 | 49.6 | Unmixed water stabilizer |
|  | 14.1 | 27.8 | 29.2 | Doped with 5% SCA-2 water stabilizer |

**Table.14 Mixed embankment settlement monitoring results**

| Measurement time (Date) | | 2021.8.3 | 2021.8.5 | 2021.8.8 | 2021.8.12 | 2021.8.18 | 2021.8.25 | 2021.9.7 | 2021.9.15 |
| --- | --- | --- | --- | --- | --- | --- | --- | --- | --- |
| Measurement point A | Settlement (mm) | 3 | 3 | 1 | 0 | 0 | 0 | 0 | 0 |
| Measurement point B |  | 3 | 2 | 1 | 1 | 1 | 1 | 0 | 0 |
| Measurement point C |  | 4 | 3 | 2 | 1 | 0 | 0 | 0 | 0 |
