# Supplementary material for: Road properties of cement-phosphogypsum-red clay under dry and wet cycles
Source: PLoS One. 2024 Dec 19;19(12):e0314276. doi: 10.1371/journal.pone.0314276 (PMC11658611; doi:10.1371/journal.pone.0314276)
Supplement: Attachment — Submitted filename: azidane hind-Review.docx [file pone.0314276.s002.docx]

azidane hind
IbnTofail University
B.P. 133, 14000

Kenitra, Morocco

October 03, 2024

Editorial Board of PLOS ONE

Dear Chief Editor of PLOS ONE

**Comments to the Author**

The manuscript entitled " Research on road performance and crack extension law of cement-phosphogypsumred clay under dry and wet cycle", which submitted to PLOS ONE has been reviewed. The comments are included at the bottom of this letter.

The manuscript has scholarly importance and it seems very interesting. The subject of this manuscript seems good. Please improve the paper writing to look the PLOS ONE paper style.

• **Abstract:** Complete the methods utilized in this paper to provide a comprehensive overview of the experimental approach.

• **Introduction:** Clarify whether the global annual production is between 100 to 280 tons or 100 to 280 million tons.

• **Section 3:** Rewrite the sentence for clarity: "Table 1 presents the basic parameters of the soil samples, while Table 2 illustrates their chemical composition."

• **Section 4 Title:** Consider renaming the section to "Design of Mix Ratios."

• **Section 4:** Include a short paragraph highlighting the significance of this technique in enhancing the performance and stability of the mixtures.

• **Section 4.1:** Provide additional explanation for selecting a 5% value, emphasizing its relevance based on prior research or empirical data.

• **Section 4.2:** Use shorter sentences to convey information effectively.

• **Section 5:** Write a brief introductory paragraph summarizing the key objectives and findings of this section.

• **Section 5.1:** Give some details about curing process

• **Section 5.2:** Begin this section with a statement on the importance of conducting water stability tests. And rewrite this sentence: At the end of the dry and wet cycle, the specimen was placed in a glass cup containing a certain amount of water, and the water surface did not go over the top of the specimen for about 2~3cm; at regular intervals, photographs were taken to record the changes that occurred in the specimen.

• **Section 5.4:** Rewrite this section step-by-step with detailed explanations of each process.

• **Section 5.6 Title:** Revise the title to include all terms followed by their abbreviations. Consider dividing the section into two paragraphs: one for the Scanning Electron Microscopy (SEM) test and another for the X-ray Diffraction (XRD) test.

• **Section 5.7:** Suggest numbering each step independently to improve clarity and organization.

• **Section 6.5:** Elaborate on each reaction, detailing the inputs and outputs involved.

• **Section 6.6:** Compare your results with standard measures for similar roads and discuss the long-term implications of your findings.

• **Section 7 Introduction:** Start with a concise introductory sentence that encapsulates the main theme of this section.

• **Future Analysis:** Expand on the rationale for selecting each test and outline your future analysis plans, emphasizing the importance of this research.

The manuscript can be accepted in its current form.
