# Supplementary material for: Road properties of cement-phosphogypsum-red clay under dry and wet cycles
Source: PLoS One. 2024 Dec 19;19(12):e0314276. doi: 10.1371/journal.pone.0314276 (PMC11658611; doi:10.1371/journal.pone.0314276)
Supplement: Attachment — Submitted filename: Response to Reviewers.docx [file pone.0314276.s003.docx]

**Response to Reviewers**

**Dear Reviewers:**

Thank you for your comments on the manuscript entitled “Road properties of cement-phosphogypsum-red clay under dry and wet cycles” . The comments are very valuable and helpful for revising and improving our paper. We have studied the comments carefully and made correction accordingly. The main corrections and responses to the comments are listed as follows:

**Response to Academic Editorial Editor:**

1. **Please ensure that your manuscript meets PLOS ONE's style requirements, including those for file naming.**

**Response:**

Thank you very much for reviewing our manuscript and for your valuable comments. We have carefully checked and revised the manuscript thoroughly against PLOS ONE's submission guidelines and style requirements.

2. **In your Methods section, please provide additional information regarding the permits you obtained for the work. Please ensure you have included the full name of the authority that approved the field site access and, if no permits were required, a brief statement explaining why.**

**Response:**

The tests performed in this paper were conducted with permission, and the laboratory was the Experimental Center of the School of Civil Engineering, Guizhou University.

3. **We suggest you thoroughly copyedit your manuscript for language usage, spelling, and grammar.**

**Response:**

Thank you for your valuable and thoughtful comments. We have carefully checked and improved the English writing in the revised manuscript.

4. **Thank you for stating the following financial disclosure: [Guizhou Provincial Science and Technology Program (Qiankehe Basic-ZK [2023] Key 016); Guiyang Municipal Science and Technology Program Project (Zhuke Contract [2024]-1-12).]. Please state what role the funders took in the study. If the funders had no role, please state: ""The funders had no role in study design, data collection and analysis, decision to publish, or preparation of the manuscript."" If this statement is not correct you must amend it as needed. Please include this amended Role of Funder statement in your cover letter; we will change the online submission form on your behalf.**

**Response:**

The funders had no role in study design, data collection and analysis, decision to publish, or preparation of the manuscript

5. **Thank you for stating the following in the Acknowledgments Section of your manuscript:**

**Response:**

Any funding-related text has been removed from the manuscript. Our funding statement has not been updated and remains

Guizhou Provincial Science and Technology Program (Qiankehe Basic-ZK [2023] Key 016); Guiyang Municipal Science and Technology Program Project (Zhuke Contract [2024]-1-12).

6. **We note that you have indicated that there are restrictions to data sharing for this study.**

**Response:**

Data has been provided as part of this article

7. **We note that your Data Availability Statement is currently as follows: [All relevant data are within the manuscript and its Supporting Information files.]**

**Response:**

All relevant data are within the manuscript and its Supporting Information files.

8. **Please include your tables as part of your main manuscript and remove the individual files. Please note that supplementary tables (should remain/ be uploaded) as separate ""supporting information"" files.**

**Response:**

We table as part of the main manuscript and remove individual documents.

**Response to reviewers:**

**Reviewer #1:**

1. **Writing Quality: The paper is poorly written and requires significant improvement in terms of language clarity and structure. The current writing lacks coherence, making it difficult for readers to follow the study's objectives, methodology, and conclusions. A thorough revision is needed to enhance the readability and presentation of the research.**

**Response:**

We thank the reviewers for their comments.

We have adjusted and improved the structure, language, coherence and grammar of the paper and manuscript based on your comments.

2. **Selection of Tests: The chosen tests, namely California Bearing Ratio (CBR) and Unconfined Compressive Strength (UCS), do not align well with the primary focus of the manuscript. While these tests provide some insight into the mechanical properties, they do not comprehensively address the broader engineering behavior of cement-phosphogypsum-red clay mixtures under dry and wet cycles. The study would benefit from the inclusion of additional relevant tests to assess long-term performance and durability.**

**Response:**

We thank the reviewers for their comments.

In our paper, we used a wet and dry cycling test protocol (Fig. 3) to simulate the long-term performance and durability of the mixes, analyzed the relationship between the unconfined compressive strength and the wet and dry cycling coefficients as well as the age (Figs. 6 and 7), and conducted test pavement paving and monitoring (Figs. 4 and 14) to further validate the performance of cement-phosphogypsum-red clay roadway under wet and dry cycling. Specific test results are presented in the paper.

**Fig.3 Schematic diagram of the wet/dry cycle process**

|  |  |  |  |
| --- | --- | --- | --- |
| (a)Survey and lay lines | (b) Ground treatment | (c) Anti-drainage treatment | (d) Mix and stir |
|  |  |  |  |
| (e) Transportation paving | (f) Flatten out | (g) Compacting | (h) Curing |

**Fig.4** **Roadbed construction process**

|  |  |
| --- | --- |
| **（a） C: P: T=5: 47.5: 47.5, Unmixed water stabiliser** | **（b） C: P: T=5: 47.5: 47.5, Addition of water stabiliser** |

**Fig.6 Relationship between unconfined compressive strength and number of wet and dry cycles**

|  |  |
| --- | --- |
| **（a） C: P: T=5: 47.5: 47.5，Unmixed water stabiliser** | **（b） C: P: T=5: 47.5: 47.5，Addition of water stabiliser** |

**Fig.7 Relationship between unconfined compressive strength and age**

|  |  |
| --- | --- |
| （a）Plot of settlement versus number of measurements | （b）Pavement appearance inspection |

**Fig.14 Test Road Monitoring Results**

3. **Experimental Design: The number of tests performed, and the selection of parameter values seem inadequate and may not fully represent the material behavior under varying conditions. A more rigorous and detailed experimental plan should be developed to ensure that the conclusions drawn are robust and generalizable.**

**Response:**

We thank the reviewers for their comments.

The number of tests we carry out wet and dry cycle 5 times, the reason is that according to the results of Figure 6, the mixture unconfined compressive strength will be reduced with the increase in the number of wet and dry cycle, and the first three wet and dry cycle mixture unconfined compressive strength decay is larger, and thereafter the decay of the flat. Therefore, only 5 times wet and dry cycle test.

The parameters of the test we considered the unconfined compressive strength, CBR value and water stabilization properties of the mix and revealed them mechanistically, which are very important road performance indicators, and carried out test road paving and monitoring to further validate the cement-phosphogypsum-red clay road performance. Of course, the next step is that we are carrying out more indicators to demonstrate.

We have added a flowchart of the experimental process.

Fig.2 Flowchart of the testing process

4. **Results Presentation: The presentation of results is suboptimal. The data lacks clarity, and the visual representation of findings (e.g., graphs, tables) does not effectively communicate the key outcomes of the study. A more structured and comprehensive approach is needed to present the experimental data in a way that enhances the understanding of the results.**

**Response:**

We thank the reviewers for their comments. We have supplemented the text with a table of specific values so that the experimental data can be presented more visibly.

**Tab.9 C: P: T=5: 47.5: 47.5, Numerical value of unconfined compressive strength without water stabilizers/ MPa**

| Number of wet and dry cycles/N | 7d curing period | 14d curing period | 28d curing period |
| --- | --- | --- | --- |
| 0 dry wet cycles | 1.51 | 2.33 | 2.67 |
| 1 dry wet cycle | 1.22 | 1.61 | 1.88 |
| 2 dry wet cycles | 0.69 | 1.18 | 1.48 |
| 3 dry wet cycles | 0.66 | 0.73 | 1.02 |
| 4 dry wet cycles | 0.58 | 0.62 | 0.87 |
| 5 dry wet cycles | 0.31 | 0.41 | 0.53 |

**Tab.10 C: P: T=5: 47.5: 47.5, Unconfined compressive strength values of water-doped stabilizers/ MPa**

| Number of wet and dry cycles/N | 7d curing period | 14d curing period | 28d curing period |
| --- | --- | --- | --- |
| 0 dry wet cycles | 0.92 | 1.21 | 1.35 |
| 1 dry wet cycle | 0.71 | 0.92 | 1.07 |
| 2 dry wet cycles | 0.61 | 0.73 | 0.81 |
| 3 dry wet cycles | 0.45 | 0.61 | 0.69 |
| 4 dry wet cycles | 0.42 | 0.53 | 0.62 |
| 5 dry wet cycles | 0.38 | 0.47 | 0.54 |

**Tab.13 Mix CBR value/%**

| Compaction degree | 7d curing period | 14d curing period | 28d curing period | Note |
| --- | --- | --- | --- | --- |
| 96% compaction degree | 25.1 | 45.9 | 49.6 | Unmixed water stabilizer |
|  | 14.1 | 27.8 | 29.2 | Doped with 5% SCA-2 water stabilizer |

5. **Discussion and Comparison with Previous Studies: The discussion section is underdeveloped and fails to critically compare the study's findings with relevant literature. A thorough comparison with previous studies is essential to establish the contribution of this research to the existing body of knowledge. The authors should strengthen this section by engaging in a more detailed analysis of how their results align or differ from previous works in the field.**

**Response:**

We thank the reviewers for their comments. We have critically discussed the findings with the relevant literature.

Section 6.1: “It was also verified in the study of Ren [39] and Cui [40] that the unconfined compressive strength of modified red clay decreased gradually with the increase of the number of wet and dry cycles, and leveled off after the third cycle. This paper further confirmed that the strength properties of the highly doped phosphogypsum stabilized materials meet the strength requirements, which reduces the damage of the phosphogypsum pollution of the environment to a certain degree, and solves the problem of the low-doped phosphogypsum road base layer material. It is difficult to meet the problem of large-scale consumption of phosphogypsum demand, and it is feasible for subsequent research.”.

Section 6.1: “According to Luo [37], it also shows that the compressive strength of the mix increases with age. With the increase of age, the hydration reaction of cement continues, and the generated gel substances such as hydrated calcium silicate and the mix undergoes cementation, the structural stability is enhanced, and the compressive strength is increased.”.

Section 6.2: “According to Peng et al. [42] showed that the coefficient of water stability of the mix decreases with the increase of the number of wet and dry cycles, due to the wet expansion and dry contraction will produce cracks, the collodion material in the specimen is damaged, and thus the strength decreases leading to the decrease of water stability.”.

Section 6.3: “According to the research results of Chen et al. [44] on the CBR properties of cement-phosphogypsum-stabilized red clay, the accuracy of the present conclusions is more confirmed. For cement-phosphogypsum-red clay used as fill for road beds on highway subgrade for Class I roads, not only the cost of cement is saved, but phosphogypsum is also highly used and the ecological environment is better protected.”.

Section 6.4: “According to the research of Gaoguo Ke [45], it can be seen that in order to verify the road performance of phosphogypsum, a test road study can be carried out to explore the feasibility of the mixture in the actual project and to detect the road performance, and this research method only measured the flatness and strength of the test road to detect the road performance. According to Liu Chao's research [12] on cement phosphogypsum stabilized crushed stone base layer, it was detected through the test road to meet the basic design requirements of high grade highway.”.

**Reviewer #2:**

1. **The abstract section should be improved to highlight the methodology, as well the objectives of the study.**

**Response:**

We thank the reviewers for their comments. We have revised the abstract and highlighted the research methodology and objectives in the abstract.

“Abstract:In this paper, the road performance and mechanism of cement-phosphogypsum-red clay (CPRC) under dry and wet cycling were systematically investigated using 5% cement as curing agent, the mass ratio of phosphogypsum: red clay = 1:1, and 5% SCA-2 as water stabilizer. The road performance of dry and wet cycle mix was verified with the National Highway G210 Duyun Yangan to Yingshan Highway Reconstruction and Expansion Project as a test road to provide a scientific basis for the application of cement-phosphogypsum-red clay on roads. The results show that the cement-phosphogypsum-red clay unconfined compressive strength decreases with the increase of the number of wet and dry cycles, with a larger decay in the first three times and leveling off thereafter. The CBR value meets the requirements of roadbed fill on highway and Class I roads as stipulated in the Design Code for Highway Roadbeds (JTG D30-2015). SCA-2 water stabilizer reduces the strength of the mixture, but significantly improve the water stability performance of the mixture, the reason SCA-2 water stabilizer active ingredients and mixing particles of physicochemical reaction, the content of ettringite in the mixture is lower than the content of the mixture not mixed with water stabilizers, the generation of quartz, white mica, and kaolinite and other hydrophilic poor material increases, the surface of the cementitious material increases, the seepage channel is reduced, so the strength is reduced, and water stability performance is improved. The roadbed of the test road was smooth and dense, with no scouring, peeling or cracking. The settlement of the roadbed is less than the requirement of “Highway Roadbed Design Specification” (JTGD30-2015). Cement-phosphogypsum-red clay (cement 5%, phosphogypsum 47.5%, red clay 47.5%, SCA-2 water stabilizer 5%) can be used as filler for road bed on highway and primary road base.”.

2. **Authors needs to precise the novelty of this work in the introduction section.**

**Response:**

We thank the reviewers for their comments. We have already described the innovation of this paper in the introduction section.

**“**Through the above research, it can be seen that the existing phosphogypsum materials used in the roadbed pavement, limited to the phosphogypsum mixed with other materials (soil, gravel, sand, fly ash), play an auxiliary role rather than as the main part of the use of the utilization rate is low (generally no more than 15%), and can not be a good solution to the problem of phosphogypsum large amounts of accumulation. In this paper, the road performance of cement-phosphogypsum-red clay (CPRC) under dry and wet cycling was investigated by using 5% cement as curing agent with the mass ratio of phosphogypsum: red clay = 1:1, plus 5% SCA-2 as water stabilizer, through the unconfined compressive strength test, the California bearing ratio (CBR) test, and the water stability test. The water stabilization mechanism of the mix was analyzed by Scanning Electron Microscope and X-ray diffraction tests. The road performance of dry and wet cycle mix was verified with the National Highway G210 Duyun Yangan to Yingshan Highway Reconstruction and Expansion Project as a test road to provide a scientific basis for the application of cement-phosphogypsum-red clay on roads. The significance of this is to improve the engineering properties of red clay while also improving the low utilization of phosphogypsum globally, reducing the pollution of phosphogypsum to the environment, and from the economic point of view, saving the cost of cement, providing a theoretical basis for the application of cement-phosphogypsum-red clay in road engineering.**”.**

3. **The experimental program (i.e., the materials and methods section) is not well-presented. It is difficult for readers to grasp the experimental setups easily. Revision is needed.**

**Response:**

We thank the reviewers for their comments.

We supplemented the test flow chart, firstly raw material test (red clay, cement, phosphogypsum We supplemented the test flow chart, firstly raw material test (red clay, cement, phosphogypsum, SCA-2) → proportion design → sample preparation → dry and wet cycle test → road performance test (CBR, unconfined compressive strength, water stabilization) → mechanistic analysis (SEM, XRD) → paving of test road and monitoring → proposing the optimal mix ratio. The whole test process is clear at a glance.

And some details of the test method are added.

“The specimens were prepared in accordance with the method of section 4.2, and after preparation, they were directly placed in the curing box at a temperature of 20±2℃ and humidity of ≥95% for 7 days, 14 days and 28 days.”.

4. **Please provide a flow chart (picture of specimens) illustrating the experimental procedures.**

**Response:**

We thank the reviewers for their comments. We have added a flowchart of the experimental process.

Fig.2 Flowchart of the testing process

5. **Discussion : Please compare your results with those previously published.** **You reported data directly without any scientific interpretation. Add a more convincing explanation to support this.**

**Response:**

We thank the reviewers for their comments. We have compared the results of the discussion with previously published results.

Section 6.1: “It was also verified in the study of Ren [39] and Cui [40] that the unconfined compressive strength of modified red clay decreased gradually with the increase of the number of wet and dry cycles, and leveled off after the third cycle. This paper further confirmed that the strength properties of the highly doped phosphogypsum stabilized materials meet the strength requirements, which reduces the damage of the phosphogypsum pollution of the environment to a certain degree, and solves the problem of the low-doped phosphogypsum road base layer material. It is difficult to meet the problem of large-scale consumption of phosphogypsum demand, and it is feasible for subsequent research.”.

Section 6.1: “According to Luo [37], it also shows that the compressive strength of the mix increases with age. With the increase of age, the hydration reaction of cement continues, and the generated gel substances such as hydrated calcium silicate and the mix undergoes cementation, the structural stability is enhanced, and the compressive strength is increased.”.

Section 6.2: “According to Peng et al. [42] showed that the coefficient of water stability of the mix decreases with the increase of the number of wet and dry cycles, due to the wet expansion and dry contraction will produce cracks, the collodion material in the specimen is damaged, and thus the strength decreases leading to the decrease of water stability.”.

Section 6.3: “According to the research results of Chen et al. [44] on the CBR properties of cement-phosphogypsum-stabilized red clay, the accuracy of the present conclusions is more confirmed. For cement-phosphogypsum-red clay used as fill for road beds on highway subgrade for Class I roads, not only the cost of cement is saved, but phosphogypsum is also highly used and the ecological environment is better protected.”.

Section 6.4: “According to the research of Gaoguo Ke [45], it can be seen that in order to verify the road performance of phosphogypsum, a test road study can be carried out to explore the feasibility of the mixture in the actual project and to detect the road performance, and this research method only measured the flatness and strength of the test road to detect the road performance. According to Liu Chao's research [12] on cement phosphogypsum stabilized crushed stone base layer, it was detected through the test road to meet the basic design requirements of high grade highway.”.

6. **Add a discussion section before conclusion regarding practical implementation of current study like the below references:**

**Concerning the practical implications, please compare your findings with existing references for conventional materials like ASTM and RILEM.**

**Response:**

We thank the reviewers for their comments. We have already compared the differences and innovations of the current study with previous conventional materials in the concluding part of the article.

“According to the research of Gaoguo Ke [45], it can be seen that in order to verify the road performance of phosphogypsum, a test road study can be carried out to explore the feasibility of the mixture in the actual project and to detect the road performance, and this research method only measured the flatness and strength of the test road to detect the road performance. According to Liu Chao's research [12] on cement phosphogypsum stabilized crushed stone base layer, it was detected through the test road to meet the basic design requirements of high grade highway.”.

At the end of the paper, we have added a discussion to analyze the reasons why phosphogypsum has not yet opened the market as a road construction material and has not achieved engineering and large-scale application, and pointed out the future research direction. Our research has been practically applied in the project, the test road is located at K35+146 of National Highway G210 Duyun Yang'an to Yingshan Highway Reconstruction and Expansion Project, adopting the standard of first-class highway, with the design speed of 60km/h, the paving length of 100m, the width of 3m, and the compaction height of 0.9m.

7. **The environmental feasibility section is not well-presented and requires major revision. It is suggested to adopt Life Cycle Assessment for better clarity.**

**Response:**

We thank the reviewers for their comments.

In the environmental feasibility part, we carried out heavy metal and radioactivity testing of phosphogypsum, and the test results showed that the heavy metal content of phosphogypsum did not exceed the relevant provisions of the national standard (GB 5085.3-2007), and the radioactivity index did not exceed the relevant provisions of the national standard (GB 6566-2010). And we tested the leachate of phosphogypsum in the field test road. At the end of the paper, we also pointed out that the reasons and future research direction of phosphogypsum have not yet opened up the market, and have not realized the engineering and large-scale application of phosphogypsum, and that the environmental protection of phosphogypsum as a road construction material is worth exploring in depth.

**Tab.5 Test Results of Heavy Metals and Radioactivity in Phosphogypsum**

| Test items | | Standard limits | Result | Conclusion |
| --- | --- | --- | --- | --- |
| Heavy metal | Cu/mg‧L^-1^ | ≤100 | 0.157 | Qualified |
|  | Zn/mg‧L^-1^ | ≤100 | 0.051 | Qualified |
|  | Cd/mg‧L^-1^ | ≤1 | 0 | Qualified |
|  | Pb/mg‧L^-1^ | ≤5 | 0 | Qualified |
|  | Cr/mg‧L^-1^ | ≤15 | 0 | Qualified |
|  | As/mg‧L^-1^ | ≤5 | 0.0356 | Qualified |
|  | Hg/mg‧L^-1^ | ≤0.1 | 0.0005 | Qualified |
| Radioactivity | Ra-226/Bq‧kg^-1^ | — | 53.94 | — |
|  | TH-232/Bq‧kg^-1^ | — | 42.13 | — |
|  | K-40/Bq‧kg^-1^ | — | 52.95 | — |
|  | I_Ra_ | ≤1.0 | 0.3 | Qualified |
|  | I_γ_ | ≤1.0 | 0.3 | Qualified |

8. **Moderate English changes are required**

**Response:**

We thank the reviewers for their comments. We have made changes to the English language.

**Reviewer #3:**

1. **Abstract: Complete the methods utilized in this paper to provide a comprehensive overview of the experimental approach.**

**Response:**

We thank the reviewers for their comments. We have made changes to the summary.

“Abstract:In this paper, the road performance and mechanism of cement-phosphogypsum-red clay (CPRC) under dry and wet cycling were systematically investigated using 5% cement as curing agent, the mass ratio of phosphogypsum: red clay = 1:1, and 5% SCA-2 as water stabilizer. The road performance of dry and wet cycle mix was verified with the National Highway G210 Duyun Yangan to Yingshan Highway Reconstruction and Expansion Project as a test road to provide a scientific basis for the application of cement-phosphogypsum-red clay on roads. The results show that the cement-phosphogypsum-red clay unconfined compressive strength decreases with the increase of the number of wet and dry cycles, with a larger decay in the first three times and leveling off thereafter. The CBR value meets the requirements of roadbed fill on highway and Class I roads as stipulated in the Design Code for Highway Roadbeds (JTG D30-2015). SCA-2 water stabilizer reduces the strength of the mixture, but significantly improve the water stability performance of the mixture, the reason SCA-2 water stabilizer active ingredients and mixing particles of physicochemical reaction, the content of ettringite in the mixture is lower than the content of the mixture not mixed with water stabilizers, the generation of quartz, white mica, and kaolinite and other hydrophilic poor material increases, the surface of the cementitious material increases, the seepage channel is reduced, so the strength is reduced, and water stability performance is improved. The roadbed of the test road was smooth and dense, with no scouring, peeling or cracking. The settlement of the roadbed is less than the requirement of “Highway Roadbed Design Specification” (JTGD30-2015). Cement-phosphogypsum-red clay (cement 5%, phosphogypsum 47.5%, red clay 47.5%, SCA-2 water stabilizer 5%) can be used as filler for road bed on highway and primary road base.”.

2. **Introduction: Clarify whether the global annual production is between 100 to 280 tons or 100 to 280 million tons.**

**Response:**

We thank the reviewers for their comments. We have made changes to the introduction.

“At present, the global annual production of phosphogypsum is 100~280 million tons, and the utilization rate of phosphogypsum is only about 15% [2].”

3. **Section 3: Rewrite the sentence for clarity: "Table 1 presents the basic parameters of the soil samples, while Table 2 illustrates their chemical composition."**

**Response:**

We thank the reviewers for their comments. We've revised the sentence.

“Table 1 presents the basic parameters of the soil samples, while Table 2 illustrates their chemical composition.”.

4. **Section 4 Title: Consider renaming the section to "Design of Mix Ratios."**

**Response:**

We thank the reviewers for their comments. We are considering renaming the title to “Design of Mix Ratios”.

5. **Section 4: Include a short paragraph highlighting the significance of this technique in enhancing the performance and stability of the mixtures.**

**Response:**

We thank the reviewers for their comments. We have already recounted the significance of the technology.

“According to previous research results [13-20], the maximum unconfined compressive strength of cemented phosphogypsum-stabilised soil was achieved when the cement dosage was 4%-6% and the ratio of cement to phosphogypsum mass was 1:1 to 1:3 [21]. Therefore, the design of the mix ratio can be carried out to effectively improve the strength properties of the mix at the lowest cost.”.

6. **Section 4.1: Provide additional explanation for selecting a 5% value, emphasizing its relevance based on prior research or empirical data.**

**Response:**

We thank the reviewers for their comments. We have revised the recommendation.

“According to Jinxiong Chen et al. [23] by studying the stabilization of red clay by cement phosphogypsum, it was concluded that the best unconfined compressive strength of the mixture can be obtained when the cement dosage is at 6%, which can meet the requirements of highway roadbed filler; According to the previous research results [24-25], when the cement is 5%, the road performance of the mixture is good and can be used as roadbed filler, too much cement will lead to shrinkage cracks in cement stabilized materials, so the cement dosage is set at 5%. In order to achieve a large amount of phosphogypsum consumption, the study of high dosage of phosphogypsum stabilized red clay road performance, set phosphogypsum: red clay = 1:1, in order to improve the water stability performance of the mixture, according to Ji Xiaoping and Huang Wendong [26-27] on the study of curing agent stabilized phosphogypsum roadbase filler, the curing agent can improve the strength and water stability of the phosphogypsum roadbase filler, so externally doped with 5% SCA-2 type water stabilizer.”.

7. **Section 4.2: Use shorter sentences to convey information effectively.**

**Response:**

We thank the reviewers for their comments. We have shortened section 4.2.

“Specimen preparation according to “highway engineering inorganic binding material stabilizer test procedures” (JTG E51-2009) [28]: need to remove the red clay and phosphogypsum in the particle size of more than 2mm particles, according to the ratio design, the optimal moisture content should be added to the calculation of the amount of water, set aside 3% of the amount of water will be the remaining water evenly mixed into the material, mixing uniformly, sealed with plastic wrap for one day, so that the mix in the water content is uniform, add the predetermined quality of cement, SCA-2 water stabilizer and the reserved water into the mix, and mix it again. The static method of compression molding: weighing the appropriate amount of mixture poured into the mold, the use of jacks to apply pressure and demolding, the preparation of specimens with a diameter and height of 50 mm.”

8. **Section 5: Write a brief introductory paragraph summarizing the key objectives and findings of this section.**

**Response:**

We thank the reviewers for their comments. We have revised the proposal.

“In this paper, the road performance of CPRC under dry and wet cycling was investigated by using 5% cement as curing agent with the mass ratio of phosphogypsum: red clay = 1:1, plus 5% SCA-2 as water stabilizer, through the unconfined compressive strength test, the CBR test, and the water stability test. The water stabilization mechanism of the mix was analyzed by Scanning Electron Microscope and X-ray diffraction test. The road performance of wet and dry recycled mixes was verified by taking the National Highway G210 Duyun Yang'an-Yingshan Highway Reconstruction and Expansion Project as a test road, which provides a scientific basis for the application of cement-phosphogypsum-red clay on roads.”.

9. **Section 5.1: Give some details about curing process**

**Response:**

We thank the reviewers for their comments. We have contained the article to make changes. Test curing is to place the finished specimen flat in the curing box, set the temperature to 20±2℃ and the humidity at ≥95% and then leave it to cure.

“The specimens were prepared in accordance with the method of section 4.2, and after preparation, they were directly placed in the curing box at a temperature of 20±2℃ and humidity of ≥95% for 7 days, 14 days and 28 days.”.

10. **Section 5.2: Begin this section with a statement on the importance of conducting water stability tests. And rewrite this sentence: At the end of the dry and wet cycle, the specimen was placed in a glass cup containing a certain amount of water, and the water surface did not go over the top of the specimen for about 2~3cm; at regular intervals, photographs were taken to record the changes that occurred in the specimen.**

**Response:**

We thank the reviewers for their comments. We have made changes.

“The roadbed is directly exposed to the atmosphere and its strength will be significantly reduced by the action of surface and groundwater. Therefore, the roadbed is not only required to have sufficient strength, but also to have sufficient water stability to keep the pavement in a normal stable condition. Therefore, in this paper, we want to study the water stability of mixes, to explore what kind of phenomenon law of its water stability.”.

“At the end of the dry and wet cycle, the specimen was placed in a glass cup containing a certain amount of water, and the water surface did not go over the top of the specimen for about 2~3cm; at regular intervals, photographs were taken to record the changes that occurred in the specimen.”.

11. **Section 5.4: Rewrite this section step-by-step with detailed explanations of each process.**

**Response:**

We thank the reviewers for their comments. We have deleted this paragraph.

12. **Section 5.6 Title: Revise the title to include all terms followed by their abbreviations. Consider dividing the section into two paragraphs: one for the Scanning Electron Microscopy (SEM) test and another for the X-ray Diffraction (XRD) test.**

**Response:**

We thank the reviewers for their comments. We have made changes.

“Scanning Electron Microscope and X-ray diffraction tests”

“The static compaction method was used to prepare the two groups of mixtures into 61.8mm, height of 20mm ring knife specimens, after preparation, they were placed in a constant temperature and humidity curing box at a temperature of (20±2)℃ and humidity of 95% for 7 days, and after 7 days, the specimens were demoulded for the scanning electron microscope test and X-ray diffraction test.

The scanning electron microscope model is HITACHI SU8100 and the accelerating voltage is 3.00kV. The ring knife specimen was broken down into soil pieces close to 2 cm³. The clods were first sanded with coarse sandpaper and then with fine sandpaper to pieces close to 0.5 cm^3^. Use clear tape to remove the powder particles from the sanding until the surface of the sample is clean. Phosphogypsum-stabilized red clay has poor electrical conductivity and requires a gold spray coating prior to scanning.

The X-ray diffractometer was a Rigaku Smart Lab with a scanning step of 0.02°, a scanning range of 15°~80°, and a scanning rate of 10 (°)/min. The test soil was the soil in the centre of the specimen.”.

13. **Section 5.7: Suggest numbering each step independently to improve clarity and organization.**

**Response:**

We thank the reviewers for their comments. We have made changes.

**“**The test road is located at K35+146 of National Highway G210 Duyun Yangan to Yingshan Highway Reconstruction and Expansion Project, adopting the standard of first-class highway, with a design speed of 60km/h, and a paving length of 100m, a width of 3m, and a compaction height of 0.9m. According to the indoor test, the proportion cement: phosphogypsum: red clay = 5 : 47.5 : 47.5, SCA-2 water stabilizer 5% was selected. The construction process is shown in Figure 4: measurement and placement → foundation treatment → anti-drainage treatment → mixing → transportation paving → leveling → rolling → maintenance.

After the roadbed paving was completed, the location 20m from both ends of the test road and the center position were selected as the settlement monitoring points (e.g., A, B, and C in Fig. 5), and the settlement of the roadbed was monitored by using a level meter. The settlement monitoring of the roadbed was measured every 5 days, totaling 7 times.**”.**

14. **Section 6.5: Elaborate on each reaction, detailing the inputs and outputs involved.**

**Response:**

We thank the reviewers for their comments. We have already clarified.

“As the phosphogypsum cement stabilized red clay in the process of dry and wet cycle, the hydration reaction of cement occurs, resulting in C-S-H gel [43], the chemical reaction formula as in equation (4), and phosphogypsum main component calcium sulfate dihydrate (CaSO_4_ • 2H_2_O), reacts with the hydration products of cement to produce ettringite, the chemical reaction formula as in equations (5) and (6).

$$\begin{aligned} 3CaO\cdot\mathrm{SiO}_{2}+nH_{2}O\to xCaO\cdot\mathrm{SiO}_{2}\cdot\left( n-3+x \right)H_{2}O+\left( 3-x \right){Ca(OH)}_{2}\#\left（ 4 \right） \end{aligned}$$

$$\begin{aligned} 3{\mathrm{Ca}\left( \mathrm{OH} \right)}_{2}+\mathrm{Al}_{2}O_{3}+\left( n-3 \right)H_{2}O\to3Ca\mathrm{Al}_{2}O_{3}\cdot nH_{2}O\#\left（ 5 \right） \end{aligned}$$

$\begin{aligned} 3CaO\cdot\mathrm{Al}_{2}O_{3}+3\left( \mathrm{CaSO}_{4}\cdot2H_{2}O \right)+2{\mathrm{Ca}\left( \mathrm{OH} \right)}_{2}+24 H_{2}O\to3 CaO\cdot\mathrm{Al}_{2}O_{3}\cdot3\mathrm{CaSO}_{4}\cdot32H_{2}O\#\left（ 6 \right） \end{aligned}$”.

15. **Section 6.6: Compare your results with standard measures for similar roads and discuss the long-term implications of your findings.**

**Response:**

We thank the reviewers for their comments. We have already clarified.

**“**According to the research of Gaoguo Ke [45], it can be seen that in order to verify the road performance of phosphogypsum, a test road study can be carried out to explore the feasibility of the mixture in the actual project and to detect the road performance, and this research method only measured the flatness and strength of the test road to detect the road performance. According to Liu Chao's research [12] on cement phosphogypsum stabilized crushed stone base layer, it was detected through the test road to meet the basic design requirements of high grade highway.”.

16. **Section 7 Introduction: Start with a concise introductory sentence that encapsulates the main theme of this section.**

**Response:**

We thank the reviewers for their comments. We have already clarified.

**“**In this paper, the road performance of CPRC under dry and wet cycling was investigated by using 5% cement as curing agent with the mass ratio of phosphogypsum: red clay = 1:1, plus 5% SCA-2 as water stabilizer, through the unconfined compressive strength test, the California bearing ratio (CBR) test, and the water stability test. The water stabilization mechanism of the mixture was analyzed by Scanning Electron Microscope and X-ray diffraction tests. The road performance of dry and wet cycle mix was verified with the National Highway G210 Duyun Yangan to Yingshan Highway Reconstruction and Expansion Project as a test road to provide a scientific basis for the application of cement-phosphogypsum-red clay on roads.**”.**

17. **Future Analysis: Expand on the rationale for selecting each test and outline your future analysis plans, emphasizing the importance of this research.**

**Response:**

We thank the reviewers for their comments. We have already clarified.

“Future Analysis:

The main ways of comprehensive utilization of phosphogypsum resources include mine filling and ecological restoration materials, road construction materials, soil conditioning agents, cement retarders, gypsum-based building materials, sulphuric acid production, chemical fillers and so on. Since 2020, desulfurization of phosphogypsum comprehensive utilization of the amount of a declining trend, comprehensive utilization of less than 50%, in a variety of ways to use, phosphogypsum used for road construction materials less than 10%, and the demand for raw materials for the huge demand for road projects, obviously the amount is too small. Restricted by the temporary absence of successful experience in large-scale application, as well as environmental protection and other administrative licenses and related policy support and other factors, phosphogypsum as a road construction material has not yet opened up the market, not to achieve engineering, large-scale application. The reasons are as follows:

(1) Lack of a clear administrative license basis, there is currently no clear environmental protection and other relevant administrative licenses as a basis.
(2) Technically feasible, but with limited transportation distance, economy to be further cost reduction, and small product coverage radius.
(3) Phosphogypsum in the road project in the application of low admixture, the accumulation of the problem is still not effectively solved, an important reason for the lack of technical support capacity, there is no formation of phosphogypsum in the road and other aspects of the systematic system of standards.

Future Research Directions: (1) Increase the basic and forward-looking technology research and development funding for the comprehensive utilization of phosphogypsum, and there is no systematic standard system covering phosphogypsum emissions, comprehensive utilization technology, comprehensive utilization products and other aspects. (2) Clarify the basis for administrative licenses. Relevant departments to clarify the environmental protection and other relevant administrative licenses as the basis for relevant policies and safeguards to guide, support and support. (3) Through the research of this paper, the phosphogypsum mixing amount reaches 47.5%, phosphogypsum is adopted as the main material of highway construction, which changes the problem of low utilization rate in the past (generally not more than 15%), not only solves the problem of phosphogypsum dumping, but also improves the engineering characteristics of the red clay, and it is recommended to popularize the application.”.

**Reviewer #4:**

1. **The paper could benefit from further explanation regarding the choice of the 5% cement and SCA-2 ratio. Why was this specific proportion chosen, and what justifies this choice for broader applications?**

**Response:**

We thank the reviewers for their comments. We have already clarified.

We have done many kinds of water stabilizer tests and found that the external mixing of 5% of SCA-2 water stabilizer is very good for improving the water stabilization performance of the mixture, so we choose 5% of SCA-2 water stabilizer.

“According to Jinxiong Chen et al. [23] by studying the stabilization of red clay by cement phosphogypsum, it was concluded that the best unconfined compressive strength of the mixture can be obtained when the cement dosage is at 6%, which can meet the requirements of highway roadbed filler; According to the previous research results [24-25], when the cement is 5%, the road performance of the mixture is good and can be used as roadbed filler, too much cement will lead to shrinkage cracks in cement stabilized materials, so the cement dosage is set at 5%. In order to achieve a large amount of phosphogypsum consumption, the study of high dosage of phosphogypsum stabilized red clay road performance, set phosphogypsum: red clay = 1:1, in order to improve the water stability performance of the mixture, according to Ji Xiaoping and Huang Wendong [26-27] on the study of curing agent stabilized phosphogypsum roadbase filler, the curing agent can improve the strength and water stability of the phosphogypsum roadbase filler, so externally doped with 5% SCA-2 type water stabilizer.”.

2. **More comparisons to similar studies or alternative materials used in roadbed construction would strengthen the argument for adopting this mixture over other options.**

**Response:**

We thank the reviewers for their comments. We have already clarified.

“Tao et al. [10] uses fly ash, water glass and quicklime to improve phosphogypsum, which dramatically improves the water stability and strength properties of phosphogypsum, and can meet the technical requirements of roadbed and even pavement sub-base; Du et al. [11] used different dosage of solid curing agent and liquid curing agent (dosage 0.5%) to improve cement phosphogypsum stabilized materials, studied the water stability properties and mechanical properties of cement phosphogypsum materials, analyzed the strength formation mechanism, and evaluated the feasibility of its use as a pavement base material; Liu et al. [12] prepared a cement-phosphogypsum stabilized crushed stone material by partially replacing fine aggregate with phosphogypsum, and the results showed that the cement dose, phosphogypsum dosage and aggregate grading all had a large effect on the strength of cement-phosphogypsum stabilized crushed stone material. Different proportioning schemes were used for the test sections of the He-Tong highway in Shaanxi province, i.e., Lime:Phosphogypsum:Soil = 5:7:88, Lime:Phosphogypsum:Soil = 6:9:85, and Lime:Phosphogypsum:Soil = 7:10:83, respectively. Through the above research, it can be seen that the existing phosphogypsum materials used in the roadbed pavement, limited to the phosphogypsum mixed with other materials (soil, gravel, sand, fly ash), play an auxiliary role rather than as the main part of the use of the utilization rate is low (generally no more than 15%), and can not be a good solution to the problem of phosphogypsum large amounts of accumulation. In this paper, the road performance of cement-phosphogypsum-red clay (CPRC) under dry and wet cycling was investigated by using 5% cement as curing agent with the mass ratio of phosphogypsum: red clay = 1:1, plus 5% SCA-2 as water stabilizer, through the unconfined compressive strength test, the California bearing ratio (CBR) test, and the water stability test. The water stabilization mechanism of the mix was analyzed by Scanning Electron Microscope and X-ray diffraction tests. The road performance of dry and wet cycle mix was verified with the National Highway G210 Duyun Yangan to Yingshan Highway Reconstruction and Expansion Project as a test road to provide a scientific basis for the application of cement-phosphogypsum-red clay on roads. The significance of this is to improve the engineering properties of red clay while also improving the low utilization of phosphogypsum globally, reducing the pollution of phosphogypsum to the environment, and from the economic point of view, saving the cost of cement, providing a theoretical basis for the application of cement-phosphogypsum-red clay in road engineering.”.

3. **The results section could provide more clarity through visual aids like more detailed graphs or tables, which would better illustrate trends in compressive strength and CBR values.**

**Response:**

We thank the reviewers for their comments.We have added specific test data that can better illustrate trends in compressive strength and CBR values.

**Tab.9 C: P: T=5: 47.5: 47.5, Numerical value of unconfined compressive strength without water stabilizers/ MPa**

| Number of wet and dry cycles/N | 7d curing period | 14d curing period | 28d curing period |
| --- | --- | --- | --- |
| 0 dry wet cycles | 1.51 | 2.33 | 2.67 |
| 1 dry wet cycle | 1.22 | 1.61 | 1.88 |
| 2 dry wet cycles | 0.69 | 1.18 | 1.48 |
| 3 dry wet cycles | 0.66 | 0.73 | 1.02 |
| 4 dry wet cycles | 0.58 | 0.62 | 0.87 |
| 5 dry wet cycles | 0.31 | 0.41 | 0.53 |

**Tab.10 C: P: T=5: 47.5: 47.5, Unconfined compressive strength values of water-doped stabilizers/ MPa**

| Number of wet and dry cycles/N | 7d curing period | 14d curing period | 28d curing period |
| --- | --- | --- | --- |
| 0 dry wet cycles | 0.92 | 1.21 | 1.35 |
| 1 dry wet cycle | 0.71 | 0.92 | 1.07 |
| 2 dry wet cycles | 0.61 | 0.73 | 0.81 |
| 3 dry wet cycles | 0.45 | 0.61 | 0.69 |
| 4 dry wet cycles | 0.42 | 0.53 | 0.62 |
| 5 dry wet cycles | 0.38 | 0.47 | 0.54 |

**Tab.13 Mix CBR value/%**

| Compaction degree | 7d curing period | 14d curing period | 28d curing period | Note |
| --- | --- | --- | --- | --- |
| 96% compaction degree | 25.1 | 45.9 | 49.6 | Unmixed water stabilizer |
|  | 14.1 | 27.8 | 29.2 | Doped with 5% SCA-2 water stabilizer |

4. **While the paper mentions the number of wet and dry cycles (up to 5), an assessment of long-term durability beyond this range would make the findings more comprehensive for real-world applications.**

**Response:**

We thank the reviewers for their comments.

The number of wet and dry tests we carry out wet and dry cycles 5 times, the reason is that according to the results in Figure 6, the mix unconfined compressive strength will be reduced with the increase in the number of wet and dry cycles, and the first three wet and dry cycles of the mix unconfined compressive strength of the decay is larger, and thereafter the decay of the decay of the smooth. Therefore, it is reasonable to express the long-term durability of the mixture by conducting only 5 wet and dry cycle tests.
